# Description and Comparison of Health Behaviors to Fitness Measures Among Boy Scouts

**DOI:** 10.7759/cureus.6262

**Published:** 2019-11-30

**Authors:** Sara K Arena, Elisabeth Bulliner, Edward Peterson

**Affiliations:** 1 Physical Therapy, Oakland University, Rochester, USA; 2 Physical Therapy, Duke University School of Medicine, Durham, USA; 3 Public Health Sciences, Henry Ford Health System, Detroit, USA

**Keywords:** boy scouts, health behaviors, fitness tests

## Abstract

The purpose of this study is to describe, compare, and correlate self-reported health behaviors (HB) to fitness test (FT) measures among boy scouts initiating the Personal Fitness Merit Badge (PFMB) requirements. A descriptive study recruited scouts ages 11-17 years using a sample of convenience. A survey of self-reported responses to physical activity, weight and nutrition, and PFMB required FTs were assessed. Descriptive statistics reported age and survey responses. A pairwise comparison examined the differences in negative and positive survey responses to FT measures; whereas the Spearman Rank correlation analyzed physical education (PE) classes and sports team participation with FT performance. Ninety-nine scouts, age 12.6 (SD 1.4) years, met inclusion criteria. Positive responses to being at a recommended weight and engaging in less screen time demonstrated significantly better performance on pull-ups, push-ups, sit-ups, and one mile run (1MR) tests; whereas scouts reporting to have been physically active at least five times in the past week performed better on the push-ups, sit-ups, and 1MR tests. Improved sit and reach flexibility and sit-up tests were correlated to more days in PE; whereas team sport participation correlated to better 1MR and sit-up test performance. Nutritional practices and reduced screen time demonstrated favorable trends among scouts compared to all-male adolescence nationally; however, weight and physical activity behaviors suggest targeted interventions are warranted. Scouts initiating the requirements of the PFMB may be well-positioned to embark on targeted health behavior changes aimed at achieving long-term healthful lifestyles.

## Introduction

American adolescents do not meet the recommended physical fitness and dietary recommendations that could promote current and future healthy living [1]. Health behaviors (HB), inclusive of physical activity (PA), established during adolescent years determine a young person’s health status and risk of developing chronic diseases in adulthood [2-3]. Ortega et al. found that increasing vigorous activity in adolescents resulted in positive fitness and mental health benefits and was associated with higher academic performance [4]. Therefore, it is imperative to educate and train adolescents in the constructs and implementation of healthy lifestyle choices inclusive of physical activity during this formative stage.

Healthy People 2020 established goals for American adolescents aimed toward increasing physical fitness, consuming a healthful diet, and maintaining a healthy body weight [2]. The physical fitness goals include increasing the percentage of adolescents who meet federal guidelines for muscle-strengthening and aerobic exercise. Additionally, a decrease in negative HB that contributes to PA and includes reducing the amount of time playing video games, viewing television, and spending non-academic computer time are addressed in the goals. Additionally, the Youth Risk Behavior Surveillance System (YRBSS) had the purpose of assessing and summarizing the current health status of Americans adolescents [5]. The YRBSS survey addressed many targeted adolescent health behaviors outlined in the Healthy People 2020 goals and objectives.

The Boy Scouts of America (BSA) is a national organization that challenges young people to be the best version of themselves, utilizing various aspects of its programming, Oath, and Law [6]. It is one of the largest youth organizations with more than 2.4 million youth participants, making it well-positioned to address current and future healthful behaviors in its participants [7]. It is notable that recent BSA policy changes enacted on February 1, 2019, now permit both male and female adolescents to join as active members [8]. This offers the possibility of future influence to a broader adolescent population. These changes resulted in rebranding and an associated name change for one of the BSA offered programs formally titled “Boy Scouts,” to its new title of “Scouts BSA,” which now has boy troops and girl troops. This branch of BSA programming is the mechanism by which the rank of Eagle Scout, a globally recognized accomplishment, is achieved [9].

The Personal Fitness Merit Badge (PFMB), an Eagle Rank required badge, requires scouts to conduct fitness test (FT) assessments and then carry out a 12-week self-directed exercise program and reassessment [10]. Fitness tests encompassed in the PFMB include push-ups, pull-ups, sit-ups, sit and reach flexibility test (SRF), and the one-mile run (1MR). Furthermore, scouts are encouraged to assess their body mass index (BMI), given age-appropriate normative values. Finally, the PFMB consists of positive health behavior learning requirements, including the significance of good nutrition and a proper weight control program [10].

Arena et al. investigated FT performance among boy scouts completing the PFMB and reported scouts that met the criteria of being overweight and obese demonstrated lower performance in all PFMB required FT with the exception of the SRF when compared to boy scouts with BMI categorized as normal and underweight. Furthermore, the study reported no significant relationships between the male scout’s age and the PFMB required FTs [11]. While this evidence provided a baseline of FT measures for scouts embarking on the PFMB, it did not report evidence of the associated HB that may be contributors to testing performance. An understanding of this evidence gap could serve to describe and compare self-reported HB to FT measurements in scouts [12-13]. This would be of benefit to the development or enhancement of HB education in the BSA and other youth-minded organization. Therefore, the purpose of this study is to describe, compare, and correlate self-reported HB to FT measures among boy scouts initiating the PFMB requirements.

## Materials and methods

Research design

After obtaining Oakland University Institutional Review Board approval #603927 to assure the rights of each participant were protected, a prospective descriptive study design was initiated utilizing a sample of convenience.

Sampling criteria

An anticipated 100 Boy Scouts (33-36 per year of data collection) were invited to participate in one four-hour fitness event on the campus of Oakland University. The event was conducted as a component of service-learning in a Doctor of Physical Therapy (DPT) curriculum required Health Promotion and Wellness in Physical Therapy course. Registered boy scouts, age 11-17 years, from the BSA Great Lakes Council servicing Southeast Michigan were invited to participate in one of three identical events offered in November of 2014, 2015, or 2016. As the dates for study participation are notably in advance of female opportunities for Scout BSA inclusion, this study was available only to male scouts. Boy scouts were informed of this study opportunity through council advertising and flyers distributed by local troop scoutmasters.

Scouts met inclusion criteria if they were of the male gender, 11 to 17 years of age, a registered member of the BSA, and parental or guardian permission was secured prior to the event date. Additionally, each scout had prior approval of their troop scoutmaster to begin work on their PFMB and the parent or guardian confirmed physician medical clearance was conducted within the year of participation. Scouts requiring any physical or environmental accommodation were also included. Scouts who did not assent, had previously completed the PFMB, or were absent on the event day were excluded from the study.

An anticipated 100 participants would result in a test with 0.80 power, with a two-sided 0.05 adjusted alpha level, to detect an effect size of 0.74. The effect size is defined as the ratio of the detectable difference in means to its standard deviation and the projected sample size will detect median to large effects.

Protocol

Data collection was conducted in conjunction with a PFMB service-learning event and offered scouts the opportunity to complete eight of the nine PFMB requirements. Scouts were aided in developing a 12-week exercise plan, which they then carried out independently after the data collection day. As the goals and requirements of the PFMB paralleled the objectives of the physical therapy health promotion and wellness coursework, DPT students in their third professional year of a DPT program engaged in a mutually beneficial service-learning opportunity with the scouts.

Under the direction of the course instructor, who was also a certified BSA merit badge counselor for the PFMB, DPT student data collectors were trained in testing methodology for a nine-question HB survey and the merit badge required FT [10]. The HB survey was administered to each scout at the cessation of the event and prior to delivering the education components of the badge. The details of the administration are outlined below. The FTs were performed under the supervision of a licensed physical therapist (PT). Detailed methodology for each of the PFMB required FT (push-ups, pull-ups, sit-ups, SRF, and 1MR) and measurement outcomes are reported in a separate publication [11].

Health behaviors survey

A nine-question HB survey was modified from the 2013 Center for Disease Control and Prevention (CDC) Middle School National Survey to include questions of body weight, eating breakfast, and PA (Table 1). While responses were recorded and reported for each query, questions 1, 4, 5, 6, and 7 had responses that reflected the scout’s self-reported HBs, which either aligned or did not align with the recommended national guidelines. Therefore, the responses were further coded as either a positive response (aligned with the recommended behavior) or a negative response (not aligned with the recommended behavior) with coding further detailed in Table 1 [14-16]. It is notable that while the investigators recognize “daily” PA is recommended for this age group, scouts who self-reported participating in PA five, six, or seven days a week were coded as positive responses consistent with YRBSS survey reporting [5].

**Table 1:** Health behaviors survey questions and response options Questions modified from 2013 Center for Disease Control and Prevention Middle School National Survey

Question #	Question	Responses options coded as positive	Response options coded as negative
1	How do you describe your weight?	Very underweight, Slightly underweight, about the right weight	Slightly overweight, very overweight
4	During the past 7 days, on how many days did you eat breakfast?	7 days	0 days, 1 day, 2 days, 3 days, 4 days, 5 days, 6 days
5	During the past 7 days, were you physically active for a total of at least 60 minutes per day? (Add up all the time you spent in any kind of physical activity that increased your heart rate and made you breathe hard some of the time.)	5 days, 6 days, 7 days	0 days, 1 day, 2 days, 3 days, 4 days
6	On an average school day, how many hours do you watch TV?	I do not watch TV on an average school day, Less than 1 hour per day, 1 hour per day, 2 hours per day	3 hours per day, 4 hours per day, 5 or more hours per day
7	On an average school day, how many hours do you play video or computer games or use a computer for something that is not school work? (Count time spent on things such as Xbox, PlayStation, an iPod, an iPad or another tablet, a smartphone, YouTube, Facebook or other social networking tools, and the Internet.)	I do not play video or computer games or use a computer for something that is not school work, Less than 1 hour per day, 1 hour per day, 2 hours per day	3 hours per day, 4 hours per day, 5 or more hours per day
Question #	Question	Response Options	
2	Which of the following are you trying to do about your weight?	Lose weight, Gain weight, Stay the same weight, I am not trying to do anything about my weight	
3	Have you ever gone without eating for 24 hours or more (also called fasting) to lose weight or to keep from gaining weight?	Yes, No	
8	In an average week when you are in school, on how many days do you go to physical education (PE) classes?	0 days, 1 day, 2 days, 3 days, 4 days, 5 days	
9	During the past 12 months, on how many sports teams did you play? (Count any teams run by your school or community groups.)	0 teams, 1 team, 2 teams, 3 or more teams	

Statistical analysis

Descriptive statistics reported scouts’ age and all nine HB survey question responses. A pairwise comparison analyzed differences in mean FT performance to questions containing positive and negative responses with statistical significance set at p< 0.05; whereas the Spearman correlation coefficient was used to compare school days in physical education (PE) classes and sports team participation to the mean FT performance. While scouts’ performance on each FT was available to the investigators for comparison and correlation analysis in this study, a prior publication has previously reported the descriptive analysis for each FT [11]. Therefore, FT measures are not included in the result reporting of this investigation. Statistical analysis was performed using SAS v.9.4 software for Windows (SAS Institute, Cary, North Carolina).

## Results

Demographics

Ninety-nine (99) Boy Scouts met inclusion criteria with 33 enrolled in each of the three data collection years. The scouts mean age was 12.6 (SD 1.4) years with an age range of 11-17 years.

Health behavior survey descriptive results

Table 2 reports the percentage of positive responses to recommended HB for each of the nine survey questions.

**Table 2:** Survey responses indicating positive response to recommended health behaviors

Question	Positive Response %
1. How do you describe your weight?	73%
2. Which of the following are you trying to do about your weight?	Lose 31%
	Gain 12%
	Stay 26 %
	Not trying anything 30%
3. Have you gone without eating for 24 hours or more to lose weight or keep from gaining weight?	3%
4. During the past 7 days, on how many days did you eat breakfast?	56%
5. During the past 7 days, on how many days were you physically active for a total of at least 60 minutes per day?	47%
6. On an average day, how many hours do you watch TV?	90%
7. On an average school day, how many hours do you play video or computer games or use a computer for something that is not school work?	74%
8. In an average week when you are in school, on how many days do you go to physical education class?	1.6 ±1.9 days
9. During the past 12 months, on how many sports teams did you play?	1.2±1.1 days

Fitness test performance comparison and correlation to survey responses

Table 3 reports the pairwise comparisons of FT performance to the positive and negative responses to HB survey questions. Scouts with positive self-responses to being at a recommended weight and engaging in less time with technology demonstrated significantly better performance on the pull-ups, push-ups, sit-ups, and 1MR tests when compared to scouts with negative responses; whereas scouts reporting to have been physically active for a total of at least 60 minutes per day at least five times in the past week performed significantly better on the push-ups, sit-ups, and 1MR tests. Additionally, superior 1MR times were identified in scouts who reported watching less television. 

**Table 3:** Comparison of fitness tests to positive and negative survey responses cm, centimeters; sec, seconds

	Fitness Test	Number of positive responders N=	Mean score on fitness test from positive responders (SD)	Number of negative responders N=	Mean score on fitness test from negative responders (SD)	P-Value
How do you describe your weight?	Pull-Up	72	2 (3)	27	1 (2)	0.001
	Push-Up	72	20 (10)	27	14 (11)	0.02
	Sit-Up	73	31 (8)	27	24 (8)	0.001
	Sit and Reach Flexibility Test (cm)	71	21 (11)	27	22 (10)	0.72
	One Mile Run (sec)	59	574 (134)	24	722 (160)	0.001
During the past 7 days, on how many days did you eat breakfast?	Pull-Up	55	2 (3)	44	2 (3)	0.61
	Push-Up	55	17 (11)	44	20 (10)	0.14
	Sit-Up	55	28 (9)	44	30 (8)	0.34
	Sit and Reach Flexibility Test (cm)	54	21 (10)	44	22 (11)	0.44
	One Mile Run (sec)	42	614 (176)	41	619 (136)	0.59
During the past 7 days, were you physically active for a total of at least 60 minutes per day?	Pull-Up	46	2 (3)	53	2 (3)	0.28
	Push-Up	46	22 (10)	53	15 (10)	0.002
	Sit-Up	46	33 (6)	53	25 (9)	0.001
	Sit and Reach Flexibility Test (cm)	46	23 (11)	52	20 (10)	0.08
	One Mile Run (sec)	36	551 (113)	47	667 (168)	0.002
On an average school day, how many hours do you watch TV?	Pull-Up	89	2 (3)	10	1 (1)	0.24
	Push-Up	89	19 (11)	10	13 (11)	0.08
	Sit-Up	89	29 (9)	10	28 (11)	0.97
	Sit and Reach Flexibility Test (cm)	88	21 (11)	10	19 (10)	0.37
	One Mile Run (sec)	74	601 (147)	9	741 (191)	0.02
On an average school day, how many hours do you play video or computer games or use a computer for something that is not school work?	Pull-Up	73	2 (3)	26	1 (3)	0.04
	Push-Up	73	20 (10)	26	14 (11)	0.02
	Sit-Up	73	30 (8)	26	25 (10)	0.04
	Sit and Reach Flexibility Test (cm)	72	22 (10)	26	18 (11)	0.06
	One Mile Run (sec)	58	582 (126)	25	697 (191)	0.01

Table 4 reports correlations between the number of days in PE class and increased sports team participation with a better performance on each of the PFMB FTs. The SRF and sit-up tests were correlated to more days in a PE class (0.28, 0.01 and 0.28, 0.01, respectively); whereas more team sport participation correlated to a better 1MR (-0.33, 0.002) and sit-up test (0.44, 0.001) performance.

**Table 4:** Correlation between better performance on fitness test and days in physical education and sports team play

Fitness Test	In an average week when you are in school, on how many days do you go to physical education classes? (r, p-value)	During the past 12 months, on how many sports teams did you play? (r, p-value)
Pull-Up	0.01, 0.97	0.01, 0.92
Push-Up	0.04, 0.71	0.07, 0.49
Sit-Up	0.28, 0.01	0.44, 0.001
Sit and Reach Flexibility Test (cm)	0.28, 0.01	0.08, 0.44
One Mile Run (sec)	-0.08, 0.46	-0.33, 0.002

## Discussion

The purpose of this study is to describe, compare, and correlate self-reported HB to FT measures among boy scouts initiating the PFMB requirements. When comparing HB survey responses of the scouts in this study to the responses reported in the YRBSS survey, which is inclusive of a more generalized adolescent male population, scouts self-reported similar rates of being at a recommended body weight (73% compared to 74%) [5]. Similar rates were also reported when asked if trying to lose weight (boy scouts, 31%; all adolescent males 33%).

While 7% of all adolescent males reported going 24 hours without eating in order to lose weight or keep from gaining weight, this behavior was lower among scouts (3%). Additionally, this study observed 56% of boy scouts to have eaten breakfast on each of the seven days prior to taking the survey; however, only 42% of adolescent males nationwide reported this same HB. Timlin et al. have suggested an inverse relationship between weight gain and eating breakfast in adolescents; however, significant self-reported weight differences were not identified in this study [17]. The scouts’ positive trend of food consumption, including eating breakfast and lower rates of fasting for weight loss, could be reflective of BSA role modeling, which is well-aligned with recommended nutrition guidelines. This is inclusive of targeted rank advancement and merit badge-related education on menu planning from a food pyramid guide and supervised meal preparation using safe food handling criteria prior to and during campouts [18].

Despite an expanded definition of the CDC recommendation of seven days/week of physical activity to a five to seven days/week window for analysis in this study, scouts self-reported compliance below the national PA rates for their age-matched counterparts (47% compared to 57%, respectively) [5,19]. Jang, Johnson, & Kim reported scouts who achieve Eagle rank were 58% more likely to exercise 30 minutes or more daily compared to other scouts [20]. However, Jang et al. also reported scouts who had achieved Eagle rank were only 18% more likely to do this same exercise regimen as non-scouts [20]. While scouts participating in our current study were initiating an Eagle rank required badge, none had yet achieved Eagle rank. Therefore, the findings of this study are in alignment with the reports of Jang et al. [20]. However, given that the scouts in this study voluntarily joined scouting and are participating in PFMB activities, it may suggest a readiness to accept and make positive changes to current HB. This would be supported in the constructs of the transtheoretical model of behavior change suggesting this sub-population of adolescents could be more likely to move through a series of stages to optimize their HBs [21]. Healthcare providers and public health agencies should consider themselves vital in promoting wellness in this population; however, further research with this intent is warranted.

Scouts reported television watching for less than two hours per day (90%) and less than two hours spent on a computer not doing homework per day (74%). Given the national averages for all adolescent males are reported as 75% and 58%, respectively, this finding suggests that scouts have more favorable screen time behaviors. Furthermore, a scout’s participation in PE classes and sports teams correlated to more favorable FT performance. Therefore, it may be beneficial to designate time aimed at encouraging movement and exercise within troop meetings and activities, as it could provide an additional opportunity for youth to achieve the recommended PA guidelines. Furthermore, several BSA merit badges promoting sports team and athletic participation are already available and should be encouraged.

Physical therapists (PTs) who were used to provide the interventions and collect the data in this study are a healthcare profession well-positioned to provide health promotion-focused assessments, interventions, and education [22-23]. Additionally, Pignataro suggests motivational interviewing (MI) to be one technique health care professionals can use to encourage individuals, including scouts, to play an active role in their health and wellness choices [24]. In this technique, the health care provider engages in directed conversation to assess and incorporate patient empowerment. As Black, Ingman, and Janes have reported, individuals are comfortable initiating conversations about various health topics with PTs making this profession and the MI technique a good option for addressing the primary and secondary prevention topics identified in this study [25].

The findings of this study are important, given that scouting is one of the largest organizations outside of the public and private educational systems that may contribute to adolescent HB education nationally. Such contributions are highlighted in its commitment to the required PFMB for its highest rank of Eagle Scout, in combination with promoting a multitude of other lifelong PAs. Furthermore, Scout BSA’s inclusion of females could offer future opportunities to make positive contributions to the HBs of young women as well.

Study limitations

Limitations to this study include the self-reporting nature of the HB survey as the utilization of a survey tool presents disadvantages, including but not limited to a respondent’s comfort level in providing accurate answers or those that present themselves in an unfavorable manner. Additionally, not controlling for the sample sizes of boy scouts age may present a bias for comparison and correlation. Furthermore, using a sample of convenience may have included only scouts interested in earning the PFMB and limits generalizability to all boy scouts. Finally, a scout’s effort on the HB survey and associated FTs may have impacted measurement outcomes.

Future research

Future research examining the outcomes given that scouts reported a readiness to change or by age may assist in determining if there is an optimal window to impact more positive HB choices. Additionally, a study exploring the outcomes of intervention techniques, inclusive of MI or health coaching, with scouts engaging in the PFMB requirements, would be beneficial in determining the long-term impact of these strategies in this population. Finally, conducting similar studies comparing other youth demographics, including Scout BSA girl troops or Venture scouting troops, would add to the generalizability to other populations of scout participants.

## Conclusions

Nutritional practices and reduced screen time demonstrated more favorable trends in scouts as compared to male adolescents nationally; however, weight and PA behaviors among scouts suggest targeted interventions are warranted. Scouts initiating the requirements of the PFMB may be well-positioned to embark on targeted HB changes aimed at achieving a long-term, healthful lifestyle.
